# Milder loss of insulin-containing islets in individuals with type 1 diabetes and type 2 diabetes-associated *TCF7L2* genetic variants

**DOI:** 10.1007/s00125-022-05818-y

**Published:** 2022-10-25

**Authors:** Maria J. Redondo, Sarah J. Richardson, Daniel Perry, Charles G. Minard, Alice L. J. Carr, Todd Brusko, Irina Kusmartseva, Alberto Pugliese, Mark A. Atkinson

**Affiliations:** 1 Department of Pediatrics, Baylor College of Medicine, Texas Children’s Hospital, Houston, TX USA; 2 Islet Biology Group (IBEx), Exeter Centre of Excellence for Diabetes Research (EXCEED), University of Exeter College of Medicine and Health, Exeter, UK; 3 Departments of Pathology and Pediatrics, University of Florida Diabetes Institute, Gainesville, FL USA; 4 Institute for Clinical and Translational Research, Baylor College of Medicine, Houston, TX USA; 5 Diabetes Research Institute, Department of Medicine, Division of Endocrinology, Department of Microbiology and Immunology, Miller School of Medicine, University of Miami, Miami, FL USA

**Keywords:** African American, Genetics, Heterogeneity, Insulin-containing cells, Pancreas, Phenotype, Precision medicine, Race, *TCF7L2*, Type 1 diabetes

## Abstract

**Aims/hypothesis:**

*TCF7L2* variants are the strongest genetic risk factor for type 2 diabetes. In individuals with type 1 diabetes, these variants are associated with a higher C-peptide AUC, a lower glucose AUC during an OGTT, single autoantibody positivity near diagnosis, particularly in individuals older than 12 years of age, and a lower frequency of type 1 diabetes-associated HLA genotypes. Based on initial observations from clinical cohorts, we tested the hypothesis that type 2 diabetes-predisposing *TCF7L2* genetic variants are associated with a higher percentage of residual insulin-containing cells (ICI%) in pancreases of donors with type 1 diabetes, by examining genomic data and pancreatic tissue samples from the Network for Pancreatic Organ donors with Diabetes (nPOD) programme.

**Methods:**

We analysed nPOD donors with type 1 diabetes (*n=*110; mean±SD age at type 1 diabetes onset 12.2±7.9 years, mean±SD diabetes duration 15.3±13.7 years, 53% male, 80% non-Hispanic White, 12.7% African American, 7.3% Hispanic) using data pertaining to residual beta cell number; quantified islets containing insulin-positive beta cells in pancreatic tissue sections; and expressed these values as a percentage of the total number of islets from each donor (mean ± SD ICI% 9.8±21.5, range 0–92.2).

**Results:**

Donors with a high ICI% (≥5) (*n=*30; 27%) vs a low ICI% (<5) (*n=*80; 73%) were older at onset (15.3±6.9 vs 11.1±8 years, *p*=0.013), had a shorter diabetes duration at donor tissue procurement (7.0±7.4 vs 18.5±14.3 years, *p*<0.001), a higher African ancestry score (0.2±0.3 vs 0.1±0.2, *p*=0.043) and a lower European ancestry score (0.7±0.3 vs 0.9±0.3, *p*=0.023). After adjustment for age of onset (*p=*0.105), diabetes duration (*p<*0.001), BMI *z* score (*p*=0.145), sex (*p=*0.351) and African American race (*p=*0.053), donors with the *TCF7L2* rs7903146 T allele (TC or TT, 45.5%) were 2.93 times (95% CI 1.02, 8.47) more likely to have a high ICI% than those without it (CC) (*p=*0.047).

**Conclusions/interpretation:**

Overall, these data support the presence of a type 1 diabetes endotype associated with a genetic factor that predisposes to type 2 diabetes, with donors in this category exhibiting less severe beta cell loss. It is possible that in these individuals the disease pathogenesis may include mechanisms associated with type 2 diabetes and thus this may provide an explanation for the poor response to immunotherapies to prevent type 1 diabetes or its progression in a subset of individuals. If so, strategies that target both type 1 diabetes and type 2 diabetes-associated factors when they are present may increase the success of prevention and treatment in these individuals.

**Graphical abstract:**

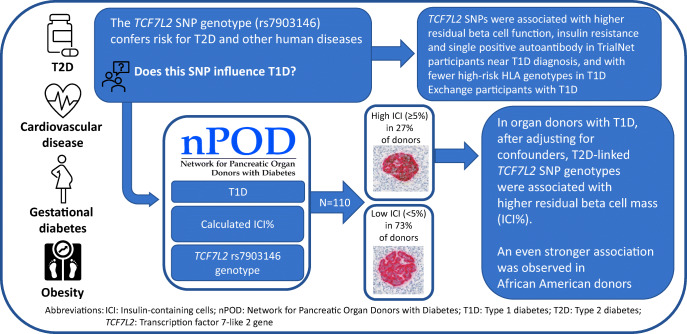

**Supplementary Information:**

The online version contains peer-reviewed but unedited supplementary material available at 10.1007/s00125-022-05818-y.

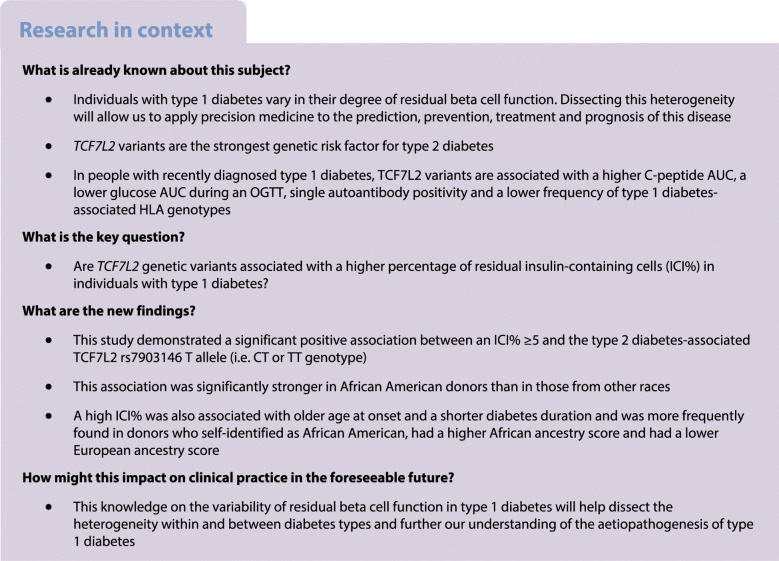



## Introduction

Type 1 diabetes is recognised as a heterogeneous condition, with emerging calls for discussion of endotypes [1]. For instance, *TCF7L2* variants—the strongest genetic risk factor for type 2 diabetes [2]—were associated with a higher C-peptide AUC, a lower glucose AUC during an OGTT and single autoantibody positivity near diagnosis, particularly in individuals older than 12 years of age, in the type 1 diabetes TrialNet study [3], and a lower frequency of type 1 diabetes-associated HLA genotypes in the T1D Exchange study [4]. Additionally, obesity/overweight accelerates the development of type 1 diabetes in high-risk individuals [5].

These data suggest that, in a subset of individuals, type 1 diabetes and type 2 diabetes-associated pathogenic mechanisms coexist and contribute to the development of diabetes. If this hypothesis is true, predictive models, diagnostic criteria and preventive and therapeutic strategies would need to account for these differences. Thus, in this study we tested the hypothesis that type 2 diabetes-predisposing *TCF7L2* SNPs are associated with a higher percentage of residual insulin-containing cells (ICI%) in pancreases of donors with type 1 diabetes by using available genomic data and tissue samples from the Network for Pancreatic Organ donors with Diabetes (nPOD) programme.

## Methods

This study included 110 nPOD donors diagnosed with type 1 diabetes and with available data on the presence/absence of residual beta cells and *TCF7L2* rs7903146 genotype. All study donors were accepted to the programme with informed consent provided by next of kin. Recovered pancreatic tissues were processed by the nPOD Organ Procurement and Pathology Core at the University of Florida (UF), as approved by the UF Institutional Review Board [6].

All nPOD donors included in this study were tested for autoantibodies to GAD65, insulinoma-associated protein 2 (IA-2A) and zinc transporter 8 using radioligand-binding assays [7, 8]. C-peptide was measured in serum samples obtained at organ recovery by electrochemiluminescence assay at UF Pathology Laboratories. Genomic DNA was isolated from spleen tissue using the Qiagen DNeasy Blood and Tissue Kit (catalogue no. 69504) and analysed for HLA genotypes at the Barbara Davis Center for Childhood Diabetes HLA Core Facility. Assessments of pancreatic pathology, endocrine cell content and extent of immune cell infiltrate were performed by routine staining for H&E, insulin, glucagon and CD3 [8].

The *TCF7L2* rs7903146 genotype was identified using a custom UFDIchip Axiom array that covers 975,000 unique variants, including those previously included on the ImmunoChip platform [9]. Genetic ancestry was imputed using a racial admixture model fit (*k=*5) to the 1000 Genomes phase 3 cohort for UFDIchip SNPs in low linkage disequilibrium.

**Fig. 1:**
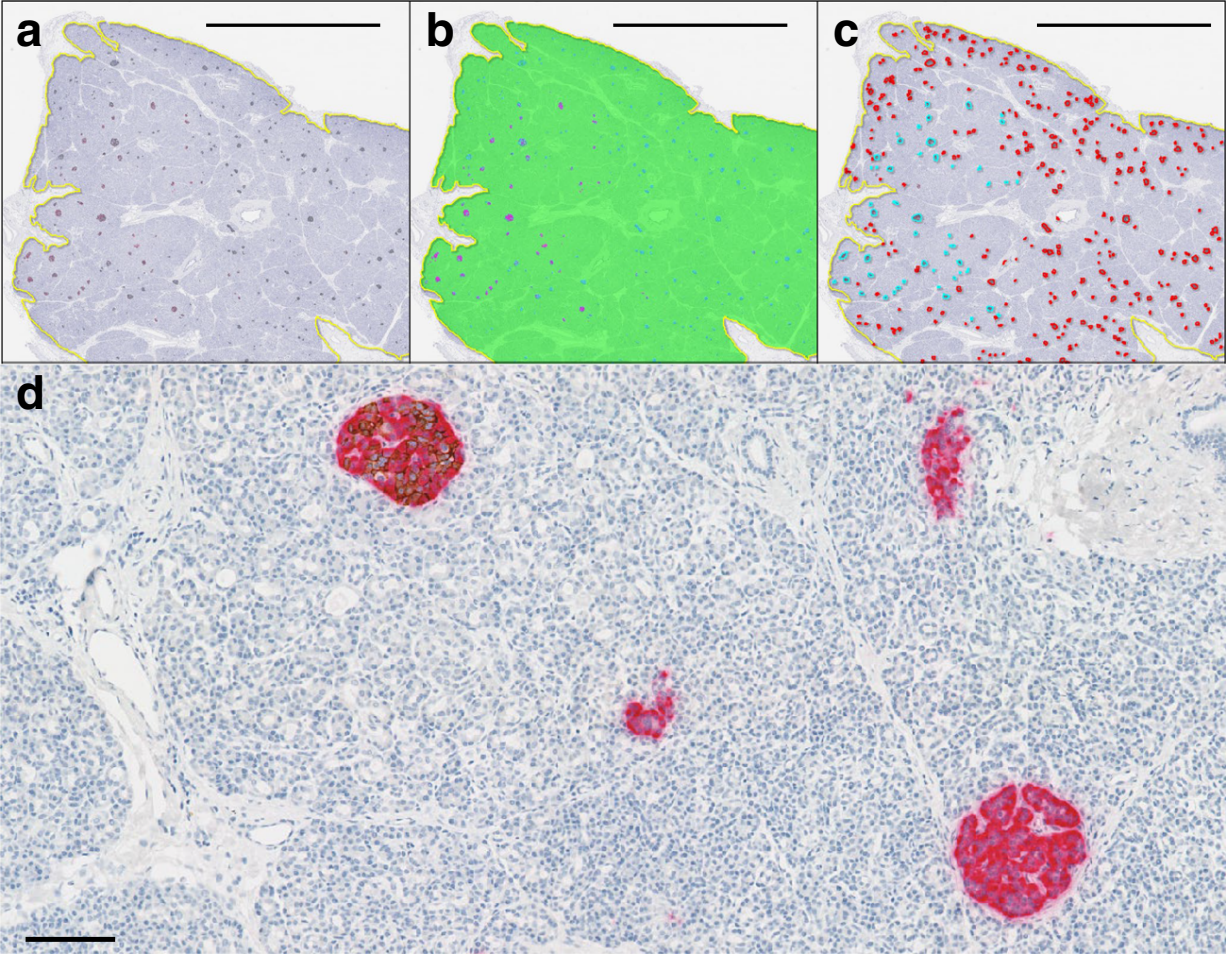
A representative section from a donor with type 1 diabetes (nPOD6228) immunostained for insulin and glucagon. (**a**) Following identification of the outer section area (yellow line), (**b**) a random forest classifier within the Indica HALO image analysis platform was used to identify insulin-positive (pink), glucagon-positive (blue) and other tissue (green) areas. (**c**) Each islet was then classified as either insulin-positive (cyan lines) or insulin-negative (red lines). Scale bar (**a**–**c**): 5 mm. The total islet count and proportion of islets that still contain insulin can then be calculated. (**d**) A higher power magnification of insulin (brown) and glucagon (pink) immunostaining in nPOD6228, demonstrating an insulin-positive islet (top left) and three insulin-negative islets. Scale bar: 100 μm

In donors with remaining beta cells, 4 μm formalin-fixed paraffin-embedded pancreatic tissue sections were stained for the presence of insulin-positive and glucagon-positive cells. Sections were double-stained with antibodies against insulin and glucagon using standard immunohistochemical approaches (Fig. 1). Sources, characteristics, dilutions and validations of primary antibodies and sources, descriptions and details of accessory agents are provided in electronic supplementary material (ESM) Table 1.

Type 1 diabetes was confirmed histologically by the presence of multiple (>10) insulin-negative islets and lobular loss of islets containing insulin-positive beta cells within the section(s) studied. Insulin-positive islet and total islet counts were completed either by standard light microscopy or using high-resolution digitised slides using the Vectra Polaris automated quantitative pathology imaging system (Akoya). ICI% was calculated by quantifying the number of islets containing insulin-positive beta cells and expressing this as a percentage of the total number of islets analysed in each donor. In the observed distribution of ICI%, the largest difference between consecutive observations under 10% occurred between 2.2 and 6.4 (ESM Fig. 1). Therefore, a high ICI% was defined as ≥5 for this study.

Statistical analyses were performed using SAS for Windows version 9.4 (Cary, NC). Donor characteristics were compared between high (≥5) and low (<5) residual ICI% groups using independent two-sample *t* tests or χ^2^ tests. Characteristics that were significantly different between groups at the 0.05 level or clinically relevant were subsequently included as predictors in a multiple logistic regression. The regression model estimated the odds of a high ICI% and the results are reported as ORs with 95% CIs. *p* values <0.05 were considered statistically significant.

## Results

Of the 110 donors included in the study, 53% were male, 80% were non-Hispanic White (NHW), 13% were African American (AA) and 7% were Hispanic (ESM Table 2). The mean age at type 1 diabetes onset was 12.2 years (range 0–36), with a mean duration of diabetes of 15.3 years (range 0–74). The type 2 diabetes-associated *TCF7L2* variant was found in homozygosis (TT genotype) or heterozygosis (TC genotype) or was absent (CC genotype) in 3.6%, 41.8% and 54.5% of the 110 donors, respectively. Overall, the mean ICI% was 9.8 (SD 21.5; range 0–92.2). There were 30 (27%) donors with a higher ICI% (i.e. ≥5) and 80 (73%) with a lower ICI% (i.e. <5).

In univariable analysis, the donors with a high residual ICI% were older at onset (15.3 vs 11.1 years, *p*=0.013), had a shorter diabetes duration (7.0 vs 18.5 years, *p*<0.001), more frequently self-identified as AA (23.3% vs 8.8%, *p*=0.041), had a higher African ancestry score (0.2 vs 0.1, *p*=0.043) and a lower European ancestry score (0.7 vs 0.9, *p*=0.023). We did not observe statistical differences between donors with higher and lower residual ICI% for sex, BMI or *TCF7L2* rs7903146 T allele (TT or TC) frequency (ESM Table 2).

Age at onset, diabetes duration, BMI *z* score, sex, AA race and *TCF7L2* rs7903146 T allele (TT/CT genotypes vs CC genotype) were included as predictors in a multiple logistic regression model. Diabetes duration (OR 0.87, *p*<0.001) and *TCF7L2* rs7903146 T allele (OR 2.93, *p*=0.047) were significantly associated with the high ICI% group (Table 1). Age at onset, AA race, BMI *z* score and sex were not statistically significantly associated with a high ICI%. A similar model was also fit including the interaction between AA race and the *TCF7L2* rs7903146 T allele, which was significant (*p=*0.0167). We observed that, among AA donors, the odds of having a higher ICI% were 182 (95% CI 4.7, 7088) times greater among donors with the T allele (i.e. TT or CT genotype) than among those without the T allele (i.e. CC genotype). Among non-AA donors, the OR was 1.69 (95% CI 0.5, 5.4).

**Table 1:** Multivariable logistic regression predicting a high ICI% group

Variable	OR	95% CI	*p* value
Age at onset^a^	1.06	0.99, 1.13	0.105
Type 1 diabetes duration	0.87	0.81, 0.94	<0.001
BMI *z* score	0.69	0.42, 1.14	0.145
Sex: female vs male	1.65	0.58, 4.74	0.351
Race: AA vs other	4.26	0.98, 18.53	0.053
rs7903146 T allele (TT, CT) vs CC	2.93	1.02, 8.47	0.047

^a^In a model with age at onset as a categorical variable (<7, 7–12 and ≥13 years), the *TCF7L2* rs7903146 T allele (TT, CT) was also statistically significant (OR 3.6, 95% CI 1.2, 11.1, *p*=0.024) compared with CC

## Discussion

We studied nPOD donors with type 1 diabetes and demonstrated a significant positive association between residual ICI%≥5 and the type 2 diabetes-associated *TCF7L2* rs7903146 T allele (i.e. CT or TT genotype). This association was significantly stronger in AA donors than in other racial groups. Unsurprisingly, shorter diabetes duration was also a predictor of residual ICI%≥5.

Overall, these data support the presence of a type 1 diabetes endotype associated with a genetic factor influencing metabolic pathways that predispose to type 2 diabetes [4], and donors in this category exhibited less severe beta cell loss. It is possible that the disease pathogenesis may include type 2 diabetes-associated mechanisms, and this may provide an explanation for the poor response to immunotherapies to prevent type 1 diabetes or its progression in a subset of individuals. If so, strategies that target both type 1 diabetes and type 2 diabetes-associated factors when they are present may increase the success of prevention and treatment.

Although the association between AA race and ICI%≥5 after adjusting for age, diabetes duration, sex, BMI and *TCF7L2* variant did not reach statistical significance (*p*=0.053), the association between *TCF7L2* variants and higher ICI% was significantly stronger in AA donors than in other races (*p=*0.0167). Potential explanations for the differences between ancestries include both genetic and environmental factors. Although genetic differences among ancestries are starting to be known, the notable type 1 diabetes genetic heterogeneity within populations of African ancestry and the influence of genetics on beta cell function warrant further study. Our knowledge on the environmental factors that affect residual beta cell function is even more limited. Obesity modifies the progression of islet autoimmunity [10] and increases the risk of type 1 diabetes [5], but this increase is almost four times higher for Hispanic than NHW autoantibody-positive adolescents and adults [11]. Although this association is yet to be tested in AA individuals, the observation that racial and ethnic minorities in the USA have a higher prevalence of obesity and, simultaneously, are experiencing higher increases in type 1 diabetes incidence than NHW individuals supports the impact of obesity on the development of type 1 diabetes. Thus, it will be important to test whether prevention of obesity lowers type 1 diabetes risk in cohorts from racially and ethnically diverse backgrounds.

Shorter diabetes duration was associated with a higher ICI%, predictably as beta cell loss continues after diabetes onset [12]. Similarly, individuals presenting at an older age have slower rates of C-peptide decline and, thus, retain higher residual beta cell function. As we adjusted for age and diabetes duration, these factors are unlikely to explain the differences seen.

Limitations of the study include its retrospective design, the use of a data-driven definition of high ICI% and that there were more than twice as many donors with a low ICI%. Additionally, our analysis was limited to nPOD donors with sufficient tissue sample for testing. While these analyses should be considered exploratory, they generate intriguing hypotheses that warrant further investigation. Studying gene expression profiles at beta cell level, along with proteomic and metabolic profiles, may further our knowledge of the mechanisms underlying our observations. Altogether, our study sheds light on the variability of residual beta cell function in type 1 diabetes. This knowledge will help dissect the heterogeneity within and between diabetes types and further our understanding of the aetiopathogenesis of type 1 diabetes.

## Supplementary information

ESM (PDF 523 kb)

## Data Availability

The datasets analysed during the current study are available from the corresponding author on reasonable request.

## Abbreviations

AA: African American
ICI%: Percentage of insulin-containing cells
NHW: Non-Hispanic White
nPOD: Network for Pancreatic Organ donors with Diabetes
UF: University of Florida

## References

[1] Battaglia M, Ahmed S, Anderson MS (2020). Introducing the endotype concept to address the challenge of disease heterogeneity in type 1 diabetes. Diabetes Care.

[2] Grant SF, Thorleifsson G, Reynisdottir I (2006). Variant of transcription factor 7-like 2 (TCF7L2) gene confers risk of type 2 diabetes. Nat Genet.

[3] Redondo MJ, Geyer S, Steck AK (2018). TCF7L2 genetic variants contribute to phenotypic heterogeneity of type 1 diabetes. Diabetes Care.

[4] Redondo MJ, Grant SFA, Davis A, Greenbaum C, T1D Exchange Biobank (2017). Dissecting heterogeneity in paediatric type 1 diabetes: association of TCF7L2 rs7903146 TT and low-risk human leukocyte antigen (HLA) genotypes. Diabet Med.

[5] Ferrara CT, Geyer SM, Liu YF (2017). Excess BMI in childhood: a modifiable risk factor for type 1 diabetes development?. Diabetes Care.

[6] Pugliese A, Yang M, Kusmarteva I (2014). The juvenile diabetes research foundation Network for Pancreatic Organ Donors with Diabetes (nPOD) program: goals, operational model and emerging findings. Pediatr Diabetes.

[7] Wasserfall C, Montgomery E, Yu L (2016). Validation of a rapid type 1 diabetes autoantibody screening assay for community-based screening of organ donors to identify subjects at increased risk for the disease. Clin Exp Immunol.

[8] Campbell-Thompson M, Wasserfall C, Kaddis J (2012). Network for Pancreatic Organ Donors with Diabetes (nPOD): developing a tissue biobank for type 1 diabetes. Diabetes Metab Res Rev.

[9] Robertson CC, Inshaw JRJ, Onengut-Gumuscu S (2021). Fine-mapping, trans-ancestral and genomic analyses identify causal variants, cells, genes and drug targets for type 1 diabetes. Nat Genet.

[10] Ferrara-Cook C, Geyer SM, Evans-Molina C (2020). Excess BMI accelerates islet autoimmunity in older children and adolescents. Diabetes Care.

[11] Tosur M, Geyer SM, Rodriguez H (2018). Ethnic differences in progression of islet autoimmunity and type 1 diabetes in relatives at risk. Diabetologia.

[12] Steck AK, Larsson HE, Liu X (2017). Residual beta-cell function in diabetes children followed and diagnosed in the TEDDY study compared to community controls. Pediatr Diabetes.

