# Supplementary material for: Milder loss of insulin-containing islets in individuals with type 1 diabetes and type 2 diabetes-associated TCF7L2 genetic variants
Source: Diabetologia. 2022 Oct 25;66(1):127–31. doi: 10.1007/s00125-022-05818-y (PMC9729318; doi:10.1007/s00125-022-05818-y)
Supplement: Supplementary file 1 — (PDF 523 kb) [file 125_2022_5818_MOESM1_ESM.pdf]

Electronic Supplementary Material (ESM)

ESM Table 1. Supplementary Tables of Reagents: Primary Antibodies (Table 1A) and Accessory Agents (Table 1B)

1A. Primary Antibodies

Primary Antibody	Manufacturer and catalogue No.	Antigen Retrieval	Antibody Dilution	Secondary Antibody Details	Validation
**Insulin**	Dako C#A0564<br>Guinea-pig polyclonal<br>(now supplied by Agilent)<br>C#IR00261-2	10mM citrate pH6.0	1/600 for 1h at RT	Dako REAL™ Envision™ Detection System, as per manufacturers instructions	Validated in pancreas tissue with appropriate negative tissue and isotype controls.<br>Used in >540 publications in the literature.<br>The antibody cross-reacts with insulin from several mammalian species. Specificity as determined by radioimmunoassay was 100% for human insulin, 100% for porcine insulin and less than 0.05% for glucagon and human growth hormone. This product has been optimised for use on human tissues.<br>Antibody Registry: AB_10013624<br>https://antibodyregistry.org/search?q=A0564
**Glucagon**	Abcam<br>C#ab10988, [K79bB10]<br>Mouse monoclonal	10mM citrate pH6.0	1/2000	Vector AP-ABC kit combined with Vector Red Substrate kit, as	Validated in pancreas tissue with appropriate negative tissue and isotype controls.

				per manufacturers instructions	Cite ab: https://www.citeab.com/antibodies/733352-ab10988-anti-glucagon-antibody-k79bb10<br>Antibody Registry: AB_297642<br>https://antibodyregistry.org/search.php?q=AB_297642

1B. Accessory Agents

Accessory Agents	Company	Catalogue No.	Description	Details
**Dako REAL peroxidase blocking system**	Dako<br>(now supplied by Agilent)	S2023<br>(S202386-2)	Blocking agent	Use as per manufacturers instructions
**Dako Antibody Diluent**	Dako<br>(now supplied by Agilent)	S2022<br>(S202230-2)	Antibody Diluent	Use as per manufacturers instructions
**Normal Goat serum**	Vector Laboratories	S1000	Blocking agent	5% NGS in Tris buffered saline
**Dako REAL™ Envision-HRP, Detection System**	Dako<br>(now supplied by Agilent)	K5007<br>(K500711-2)	Secondary detection system used for Insulin antibody	Use as per manufacturers instructions
**Vector AP-ABC kit (Mouse)**	Vector Laboratories	AK-5002	Secondary detection system used for glucagon antibody	Use as per manufacturers instructions
**Vector Red Substrate kit**	Vector Laboratories	SK-5100	Secondary detection system used for glucagon antibody	Use as per manufacturers instructions
**Dako Haematoxylin**	Dako<br>(now supplied by Agilent)	S2020	Counterstain	Use as per manufacturers instructions

ESM Table 2. Characteristics of donors in the study (n=110), and comparison by residual ICI%

	All	Residual ICI<5% N=80	Residual ICI≥5% N=30	p-value
**% residual ICI: Mean (SD) (range)**	9.8 (21.5) (0.0, 92.2)	0.2 (0.5) (0, 2.2)	35.6 (28.3) (6.4, 92.2)	<0.001
**Age, attained (years): Mean (SD) (range)**	27.9 (13.4) (4.4, 78.0)	30 (14.3) (4.4, 78.0)	22.2 (8.8) (11.0, 45.0)	0.007
**Age at T1D onset (years): Mean (SD) (range)**	12.2 (7.9) (0-36)	11.1 (8) (0.0,36.0)	15.3 (6.9) (3.0,35.0)	0.013
**Age at T1D onset category:**				0.007
**<7 years**	32 (29.1%)	30 (37.5%)	2 (6.7%)	

**7-12 years**	31 (28.2%)	20 (25.0%)	11 (36.7%)	
**≥13 years**	47 (42.7%)	30 (37.5%)	17 (56.7%)	
**T1D duration (years): Mean (SD) (range)**	15.3 (13.7) (0-74)	18.5 (14.3) (0.0,74.0)	7.0 (7.4) (0.0,32.5)	<0.001
**BMI: Mean (SD) (range)**	24.5 (4.6) (12.9-42.5)	24.7 (4.1) (15.9,37.1)	24.1 (5.8) (12.9,42.5)	0.610
**BMI percentile*: Mean (SD) (range)**	67.0 (26.3) (2.0, 98.1)	68.2 (24.2) (2.0,98.1)	63.9 (31.5) (2.0,98.1)	0.445
**BMI Z-score*: Mean (SD) (range)**	0.6 (1.0) (-3.4, 3.9)	0.6 (0.9) (-2.1,3.9)	0.4 (1.2) (-3.4,2.2)	0.316
**BMI Category**				0.455
**Underweight**	8 (7.3%)	4 (5%)	4 (13.3%)	

**Normal**	53 (48.2%)	39 (48.8%)	14 (46.7%)	
**Overweight**	32 (29.1%)	25 (31.3%)	7 (23.3%)	
**Obese**	17 (15.5%)	12 (15%)	5 (16.7%)	
**Gender**				0.227
**Female**	52 (47.3%)	35 (43.8%)	17 (56.7%)	
**Male**	58 (52.7%)	45 (56.3%)	13 (43.3%)	
**Race/ethnicity**				0.083
**Non-Hispanic White**	88 (80%)	68 (85%)	20 (66.7%)	
**African American**	14 (12.7%)	7 (8.8%)	7 (23.3%)	
**Hispanic/Latino**	8 (7.3%)	5 (6.3%)	3 (10%)	
**African American race**				0.0410

**African American**	14 (12.7%)	7 (8.8%)	7 (23.3%)	
**Other**	96 ((87.3%)	73 (91.3%)	23 (76.7%)	
**Ancestry score: Mean (SD) (range)**				
**AFR**	0.1 (0.3)<br>(0.0,0.9)	0.1 (0.2)<br>(0.0,0.9)	0.2 (0.3)<br>(0.0,0.9)	0.043
**AMR**	0.0 (0.1)<br>(0.0,0.8)	0.0 (0.1)<br>(0.0,0.8)	0.1 (0.2)<br>(0.0,0.6)	0.395
**EAS**	0.0 (0.0)<br>(0.0,0.0)	0.0 (0.0)<br>(0.0,0.0)	0.0 (0.0)<br>(0.0,0.0)	0.149
**EUR**	0.8 (0.3)<br>(0.1,1.0)	0.9 (0.3)<br>(0.1,1.0)	0.7 (0.3)<br>(0.1,1.0)	0.023
**SAS**	0.0 (0.0)	0.0 (0.0)	0.0 (0.0)	0.663

	(0.0,0.1)	(0.0,0.1)	(0.0,0.1)	
TCF7L2 **rs7903146 (Major allele: C)**				
**CC**	60 (54.5%)	47 (58.8%)	13 (43.3%)	0.322
**TC**	46 (41.8%)	30 (37.5%)	16 (53.3%)	
**TT**	4 (3.6%)	3 (3.8%)	1 (3.3%)	

*BMI percentile and Z-score for adults were obtained by assigning the age of 20 years old.

ESM Figure 1. Distribution of ICI% in the study sample

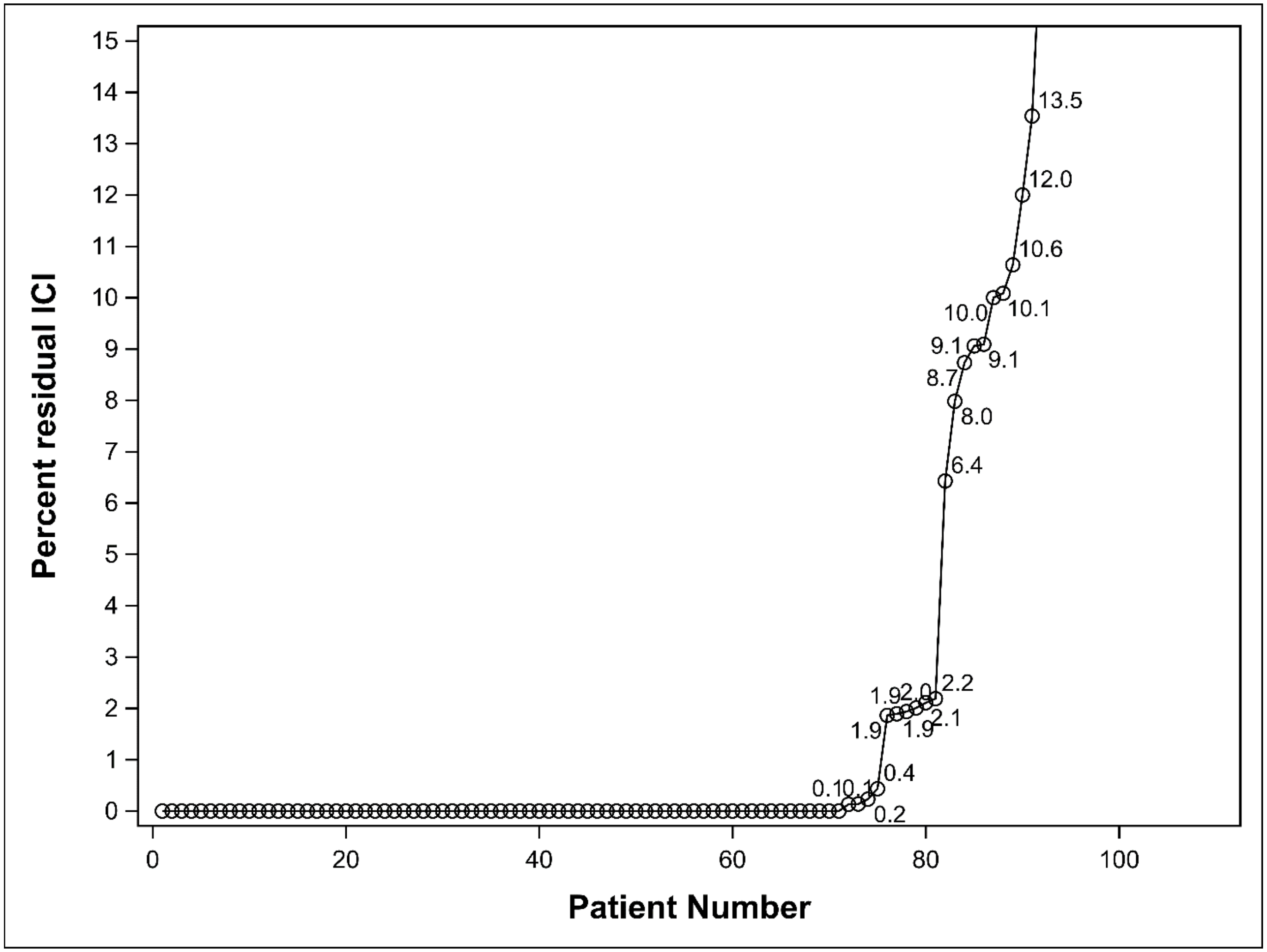

Checklist for reporting human islet preparations used in research

Diabetologia

Adapted from Hart NJ, Powers AC (2018) Progress, challenges, and suggestions for using human islets to understand islet biology and human diabetes. Diabetologia https://doi.org/10.1007/s00125-018-4772-2

(1) Authors' note: Please note that our study examined tissue sections and not isolated islets and thus, "origin/source of islets" and "isolation centre" do not apply.

**Islet preparation**	**1**	**2**	**3**	**4**	**5**	**6**	**7**	**8[a]**
**MANDATORY INFORMATION**								
Unique identifier	6025	6026	6031	6032	6035	6036	6038	6039
Donor age (years)	23.8	22.4	39	33.8	32.1	49.2	37.2	28.7
Donor sex (M/F)	Male	Male	Male	Male	Male	Female	Female	Female
Donor BMI (kg/m^2)	26.6	24.1	24.5	29.4	27.1	25.5	30.9	23.4
Donor HbA_{1c} or other measure of blood glucose control	n/a	n/a	n/a	n/a	n/a	n/a	n/a	n/a
Origin/source of islets[b] Please see authors' note: N/A								
Islet isolation centre Please see authors' note: N/A								
Donor history of diabetes? Please select yes/no from drop down list	Yes	Yes	Yes	Yes	Yes	Yes	Yes	Yes
**If Yes, complete the next two lines if this information is available**								
Diabetes duration (years)	19.00	9.00	35.00	0.00	28.00	34.00	20.00	12.00
Glucose-lowering therapy at time of death[c]	Yes: insulin	Yes: Humalog and Lantus	Yes: insulin	n/a	Yes: humulin N	Yes: insulin	Yes: Humulin 70/30	Yes: insulin
**RECOMMENDED INFORMATION**								

Donor cause of death								
Warm ischaemia time (h)								
Cold ischaemia time (h)								
Estimated purity (%)								
Estimated viability (%)								
Total culture time (h)[d]								
Glucose-stimulated insulin secretion or other functional measurement[e]								
Handpicked to purity? Please select yes/no from drop down list								
Additional notes								

[a]If you have used more than eight islet preparations, please complete additional forms as necessary
[b]For example, IIDP, ECIT, Alberta IsletCore
[c]Please specify the therapy/therapies
[d]Time of islet culture at the isolation centre, during shipment and at the receiving laboratory
[e]Please specify the test and the results

Islet preparation	9	10	11	12	13	14	15	16
**MANDATORY INFORMATION**								
Unique identifier	6040	6041	6042	6045	6046	6051	6052	6054
Donor age (years)	50	26.3	60	26.4	18.8	20.3	12	35.1
Donor sex (M/F)	Female	Male	Female	Male	Female	Male	Male	Female
Donor BMI (kg/m^2)	31.6	28.4	23.4	23.1	25.2	21.5	20.3	30.4
Donor HbA_{1c} or other measure of blood glucose control	7.3	n/a	n/a	n/a	n/a	n/a	n/a	n/a
Origin/source of islets[b] Please see authors' note: N/A								
Islet isolation centre Please see authors' note: N/A								
Donor history of diabetes? Please select yes/no from drop down list	Yes	Yes	Yes	Yes	Yes	Yes	Yes	Yes
**If Yes, complete the next two lines if this information is available**								
Diabetes duration (years)	20.00	23.00	59.00	8.00	8.00	13.00	1.00	30.00
Glucose-lowering therapy at time of death[c]	Yes: Humalog	Yes: Novalin and Lantes	n/a	Yes: OTC insulin	Yes: Lantus and Novolog	Yes: Humalog and Lantus	Yes: Humalog and Lantus	Yes: humulin N / humulin R
**RECOMMENDED INFORMATION**								
Donor cause of death								
Warm ischaemia time (h)								
Cold ischaemia time (h)								
Estimated purity (%)								
Estimated viability (%)								

Total culture time (h)[d]								
Glucose-stimulated insulin secretion or other functional measurement[e]								
Handpicked to purity? Please select yes/no from drop down list								
Additional notes								

Islet preparation	17	18	19	20	21	22	23	24
**MANDATORY INFORMATION**								

Unique identifier	6061	6063	6064	6066	6067	6070	6071	6077
Donor age (years)	28.1	4.4	19.6	78	32.6	22.6	28	32.9
Donor sex (M/F)	Male	Male	Female	Male	Female	Female	Female	Female
Donor BMI (kg/m^2)	22.1	23.8	22.6	30.9	26.8	21.6	19.5	22
Donor HbA_{1c} or other measure of blood glucose control	n/a	n/a	n/a	n/a	n/a	n/a	7.3	12.4
Origin/source of islets[b] Please see authors' note: N/A								
Islet isolation centre Please see authors' note: N/A								
Donor history of diabetes? Please select yes/no from drop down list	Yes	Yes	Yes	Yes	Yes	Yes	Yes	Yes
**If Yes, complete the next two lines if this information is available**								
Diabetes duration (years)	23.00	3.00	9.00	74.00	8.00	7.00	17.00	19.00
Glucose-lowering therapy at time of death[c]	Yes: Lantus and Novolog	Yes: Lantus and Novolog	Yes: insulin	Yes: Humalog and Lantus	Yes: insulin	Yes: Lantus (non-compliant)	Yes: Lantus and Novolog	Yes: insulin
**RECOMMENDED INFORMATION**								
Donor cause of death								
Warm ischaemia time (h)								
Cold ischaemia time (h)								
Estimated purity (%)								
Estimated viability (%)								
Total culture time (h)[d]								
Glucose-stimulated insulin secretion or other functional measurement[e]								

Handpicked to purity? Please select yes/no from drop down list								
Additional notes								

**Islet preparation**	**25**	**26**	**27**	**28**	**29**	**30**	**31**	**32**
**MANDATORY INFORMATION**								
Unique identifier	6079	6083	6084	6087	6088	6089	6113	6119
Donor age (years)	11.1	15.2	14.2	17.5	31.2	14.3	13.1	27.8
Donor sex (M/F)	Female	Female	Male	Male	Male	Male	Female	Male

Donor BMI (kg/m^2)	18.6	18.4	26.3	21.9	27	26	24.75	19.4
Donor HbA_{1c} or other measure of blood glucose control	n/a	n/a	n/a	n/a	n/a	10.4	n/a	8.4
Origin/source of islets[b] Please see authors' note: N/A								
Islet isolation centre Please see authors' note: N/A								
Donor history of diabetes? Please select yes/no from drop down list	Yes	Yes	Yes	Yes	Yes	Yes	Yes	Yes
**If Yes, complete the next two lines if this information is available**								
Diabetes duration (years)	8.00	11.00	4.00	4.00	5.00	8.00	1.58	14.00
Glucose-lowering therapy at time of death[c]	Yes: Lantus, NPH, Lispro	Yes: Humalog	Yes: insulin	Yes: Humalog and Lantus	Yes: insulin	Yes: Humalog and Lantus	Yes: insulin	Yes: Novolog
**RECOMMENDED INFORMATION**								
Donor cause of death								
Warm ischaemia time (h)								
Cold ischaemia time (h)								
Estimated purity (%)								
Estimated viability (%)								
Total culture time (h)[d]								
Glucose-stimulated insulin secretion or other functional measurement[e]								
Handpicked to purity? Please select yes/no from drop down list								

Additional notes								

**Islet preparation**	**33**	**34**	**35**	**36**	**37**	**38**	**39**	**40**
**MANDATORY INFORMATION**								
Unique identifier	6128	6141	6143	6145	6148	6150	6152	6155
Donor age (years)	33.8	36.7	32.6	18	17.1	41.2	29.6	50
Donor sex (M/F)	Female	Male	Female	Male	Male	Male	Female	Female
Donor BMI (kg/m^2)	22.2	26	26.1	23.1	23.9	25.5	30.1	26

Donor HbA_{1c} or other measure of blood glucose control	n/a	n/a	n/a	n/a	n/a	n/a	11.3	n/a
Origin/source of islets[b] Please see authors' note: N/A								
Islet isolation centre Please see authors' note: N/A								
Donor history of diabetes? Please select yes/no from drop down list	Yes	Yes	Yes	Yes	Yes	Yes	Yes	Yes
**If Yes, complete the next two lines if this information is available**								
Diabetes duration (years)	31.50	28.00	7.00	11.00	7.00	35.00	12.00	43.00
Glucose-lowering therapy at time of death[c]	Yes: insulin	Yes: Lantus and Novolog	Yes: Humalog and Lantus	Yes: insulin	Yes: Humalog	Yes: insulin	Yes: Humalog and Lantus	Yes: Novolog and Novolin N
**RECOMMENDED INFORMATION**								
Donor cause of death								
Warm ischaemia time (h)								
Cold ischaemia time (h)								
Estimated purity (%)								
Estimated viability (%)								
Total culture time (h)[d]								
Glucose-stimulated insulin secretion or other functional measurement[e]								
Handpicked to purity? Please select yes/no from drop down list								
Additional notes								

Islet preparation	41	42	43	44	45	46	47	48
**MANDATORY INFORMATION**								
Unique identifier	6159	6161	6163	6169	6173	6180	6195	6196
Donor age (years)	50.8	19.2	32.5	27.6	44.1	27.1	19.3	26.5
Donor sex (M/F)	Female	Female	Male	Female	Male	Male	Male	Female
Donor BMI (kg/m^2)	35.5	36.1	25.5	25	23.9	25.9	23.7	26.6
Donor HbA_{1c} or other measure of blood glucose control	n/a	11.1	10.1	9.5	n/a	n/a	n/a	n/a
Origin/source of islets[b] Please see authors' note: N/A								

Islet isolation centre<br>Please see authors' note: N/A								
Donor history of diabetes?<br>Please select yes/no from drop down list	Yes	Yes	Yes	Yes	Yes	Yes	Yes	Yes
**If Yes, complete the next two lines if this information is available**								
Diabetes duration (years)	44.00	7.00	30.00	15.00	15.00	11.00	5.00	15.00
Glucose-lowering therapy at time of death[c]	Yes: insulin	Yes: Humulin R	Yes: insulin	Yes: insulin	Yes: Humalog and Lantus	Yes: Novolog	Yes: insulin	Yes: insulin
**RECOMMENDED INFORMATION**								
Donor cause of death								
Warm ischaemia time (h)								
Cold ischaemia time (h)								
Estimated purity (%)								
Estimated viability (%)								
Total culture time (h)[d]								
Glucose-stimulated insulin secretion or other functional measurement[e]								
Handpicked to purity?<br>Please select yes/no from drop down list								
Additional notes								

Islet preparation	49	50	51	52	53	54	55	56
**MANDATORY INFORMATION**								
Unique identifier	6198	6204	6205	6207	6208	6209	6211	6212
Donor age (years)	22	28	40.9	16.7	32.6	5	24	20
Donor sex (M/F)	Female	Male	Female	Female	Female	Female	Female	Male
Donor BMI (kg/m^2)	23.1	22.9830484	22.6	24.4	23.4	15.9	24.4	29.1
Donor HbA_{1c} or other measure of blood glucose control	n/a	7.2	n/a	n/a	n/a	n/a	10.8	6.4
Origin/source of islets[b] Please see authors' note: N/A								
Islet isolation centre Please see authors' note: N/A								
Donor history of diabetes? Please select yes/no from drop down list	Yes	Yes	Yes	Yes	Yes	Yes	Yes	Yes

If Yes, complete the next two lines if this information is available								
Diabetes duration (years)	3.00	21.00	33.00	10.00	16.00	0.25	4.00	5.00
Glucose-lowering therapy at time of death[c]	Yes: 70/30	Yes: insulin (non-compliant most of time)	Yes: Lantus and Novolog	Yes: Humalog and Lantus	Yes: insulin	Yes: Humalog and Lantus	Yes: Lantus and Novolog	Insulin: Humalog
**RECOMMENDED INFORMATION**								
Donor cause of death								
Warm ischaemia time (h)								
Cold ischaemia time (h)								
Estimated purity (%)								
Estimated viability (%)								
Total culture time (h)[d]								
Glucose-stimulated insulin secretion or other functional measurement[e]								
Handpicked to purity? Please select yes/no from drop down list								
Additional notes								

Islet preparation	57	58	59	60	61	62	63	64
**MANDATORY INFORMATION**								
Unique identifier	6215	6220	6223	6224	6228	6231	6236	6237
Donor age (years)	34	35	61	21	13	49	25	18
Donor sex (M/F)	Male	Female	Male	Female	Male	Female	Male	Female
Donor BMI (kg/m^2)	26.6	27.4	23.4	22.8	17.36	25.4	20.1	26
Donor HbA_{1c} or other measure of blood glucose control	8	n/a	n/a	n/a	13.3	10.4	11.6	n/a
Origin/source of islets[b] Please see authors' note: N/A								
Islet isolation centre Please see authors' note: N/A								
Donor history of diabetes? Please select yes/no from drop down list	Yes	Yes	Yes	Yes	Yes	Yes	Yes	Yes
**If Yes, complete the next two lines if this information is available**								
Diabetes duration (years)	15.00	11.00	52.00	1.50	0.00	20.00	11.00	12.00

Glucose-lowering therapy at time of death[c]	Yes: insulin	Yes: insulin	Yes: insulin	Yes: Humulin and Novolog	No	Yes: Lantus and Novolog	Yes: Humalog and Lantus	Yes: insulin
**RECOMMENDED INFORMATION**								
Donor cause of death								
Warm ischaemia time (h)								
Cold ischaemia time (h)								
Estimated purity (%)								
Estimated viability (%)								
Total culture time (h)[d]								
Glucose-stimulated insulin secretion or other functional measurement[e]								
Handpicked to purity? Please select yes/no from drop down list								
Additional notes								

**Islet preparation**	**65**	**66**	**67**	**68**	**69**	**70**	**71**	**72**
**MANDATORY INFORMATION**								
Unique identifier	6241	6242	6243	6244	6245	6246	6247	6258
Donor age (years)	33	39	13	34	22	41	24	39
Donor sex (M/F)	Male	Male	Male	Male	Male	Female	Male	Female
Donor BMI (kg/m^2)	18.4	19.5	21.3	23.8	23.2	21.1	24.27	28.7
Donor HbA_{1c} or other measure of blood glucose control	n/a	7.9	13.1	5.9	n/a	13.4	n/a	8
Origin/source of islets[b] Please see authors' note: N/A								
Islet isolation centre Please see authors' note: N/A								
Donor history of diabetes? Please select yes/no from drop down list	Yes	Yes	Yes	Yes	Yes	Yes	Yes	Yes
**If Yes, complete the next two lines if this information is available**								
Diabetes duration (years)	31.00	19.00	5.00	28.00	7.00	27.00	0.60	37.00
Glucose-lowering therapy at time of death[c]	Yes: Humalog	Yes: Humalog	Yes: Novolog	Yes: insulin	Yes: Humalog and Lantus	Yes: insulin	Yes: Lantus and Novolog	Yes: insulin
**RECOMMENDED INFORMATION**								
Donor cause of death								

Warm ischaemia time (h)								
Cold ischaemia time (h)								
Estimated purity (%)								
Estimated viability (%)								
Total culture time (h)[d]								
Glucose-stimulated insulin secretion or other functional measurement[e]								
Handpicked to purity? Please select yes/no from drop down list								
Additional notes								

Islet preparation	73	74	75	76	77	78	79	80
**MANDATORY INFORMATION**								
Unique identifier	6261	6262	6264	6265	6266	6268	6281	6285
Donor age (years)	16	44	12	11	30	12	37	22
Donor sex (M/F)	Male	Male	Female	Male	Male	Female	Male	Female
Donor BMI (kg/m^2)	20.7	21.5	22	12.85	27.1	26.6	37.1	21.8
Donor HbA_{1c} or other measure of blood glucose control	7.2	n/a	8.9	n/a	13	9.8	7.6	n/a
Origin/source of islets[b] Please see authors' note: N/A								
Islet isolation centre Please see authors' note: N/A								
Donor history of diabetes? Please select yes/no from drop down list	Yes	Yes	Yes	Yes	Yes	Yes	Yes	Yes
**If Yes, complete the next two lines if this information is available**								
Diabetes duration (years)	14.17	8.00	9.00	8.00	23.00	3.00	20.00	15.00
Glucose-lowering therapy at time of death[c]	Yes: Novolog and Humalog	Yes: Novolog and Levemir	Yes: Humalog, Novolog, Lantus	Yes: insulin	Yes: insulin	Yes: Novolog	Yes: Humalog and Lantus	Yes: insulin
**RECOMMENDED INFORMATION**								
Donor cause of death								

Warm ischaemia time (h)								
Cold ischaemia time (h)								
Estimated purity (%)								
Estimated viability (%)								
Total culture time (h)[d]								
Glucose-stimulated insulin secretion or other functional measurement[e]								
Handpicked to purity? Please select yes/no from drop down list								
Additional notes								

Islet preparation	81	82	83	84	85	86	87	88
**MANDATORY INFORMATION**								
Unique identifier	6296	6298	6299	6302	6306	6307	6319	6321
Donor age (years)	20	29	32	38.5	19	45	52	27
Donor sex (M/F)	Male	Male	Male	Male	Male	Female	Male	Female
Donor BMI (kg/m^2)	32	24.3	31.8	20.5	24.5	19.48	25.5	20.3
Donor HbA_{1c} or other measure of blood glucose control	5.6	13.3	n/a	8.2	10.1	n/a	8.6	8.1
Origin/source of islets[b] Please see authors' note: N/A								
Islet isolation centre Please see authors' note: N/A								
Donor history of diabetes? Please select yes/no from drop down list	Yes	Yes	Yes	Yes	Yes	Yes	Yes	Yes
**If Yes, complete the next two lines if this information is available**								
Diabetes duration (years)	12.00	26.00	23.00	32.50	5.00	10.00	25.00	16.00
Glucose-lowering therapy at time of death[c]	Yes: insulin	Yes: insulin	Yes: insulin	Yes: Lantus	Yes: Lantus and Novolog	Yes: insulin	Yes: insulin	Yes: Humalog
**RECOMMENDED INFORMATION**								
Donor cause of death								
Warm ischaemia time (h)								
Cold ischaemia time (h)								
Estimated purity (%)								
Estimated viability (%)								

Total culture time (h)[d]								
Glucose-stimulated insulin secretion or other functional measurement[e]								
Handpicked to purity? Please select yes/no from drop down list								
Additional notes								

**Islet preparation**	**89**	**90**	**91**	**92**	**93**	**94**	**95**	**96**
**MANDATORY INFORMATION**								
Unique identifier	6322	6323	6325	6327	6328	6330	6337	6341

Donor age (years)	22	22	20	71.2	39	22	20.6	26
Donor sex (M/F)	Male	Female	Female	Male	Male	Male	Female	Male
Donor BMI (kg/m^2)	23.6	24.7	31.2	23.2	23.98	22.6	17.9	21.8
Donor HbA_{1c} or other measure of blood glucose control	n/a	6.6	n/a	n/a	8.7	n/a	12.5	13.6
Origin/source of islets[b] Please see authors' note: N/A								
Islet isolation centre Please see authors' note: N/A								
Donor history of diabetes? Please select yes/no from drop down list	Yes	Yes	Yes	Yes	Yes	Yes	Yes	Yes
**If Yes, complete the next two lines if this information is available**								
Diabetes duration (years)	17.00	6.00	6.00	57.00	20.00	18.00	5.00	15.00
Glucose-lowering therapy at time of death[c]	Yes: insulin	Yes: Novolog	Yes: insulin	Yes: insulin	Yes: insulin	Yes: Novolog	Yes: insulin	Yes: insulin
**RECOMMENDED INFORMATION**								
Donor cause of death								
Warm ischaemia time (h)								
Cold ischaemia time (h)								
Estimated purity (%)								
Estimated viability (%)								
Total culture time (h)[d]								
Glucose-stimulated insulin secretion or other functional measurement[e]								
Handpicked to purity? Please select yes/no from drop down list								

Additional notes								

Islet preparation	97	98	99	100	101	102	103	104
**MANDATORY INFORMATION**								
Unique identifier	6342	6360	6362	6367	6371	6380	6396	6399
Donor age (years)	14	4.8	24.9	24	12.5	11.6	17.1	17.42
Donor sex (M/F)	Female	Female	Male	Male	Female	Female	Female	Male
Donor BMI (kg/m^2)	24.3	26.1	28.5	25.7	16.6	14.6	22.6	32

Donor HbA_{1c} or other measure of blood glucose control	9.2	10.2	10	8.8	9.5	13.5	13.4	10.4
Origin/source of islets[b] Please see authors' note: N/A								
Islet isolation centre Please see authors' note: N/A								
Donor history of diabetes? Please select yes/no from drop down list	Yes	Yes	Yes	Yes	Yes	Yes	Yes	Yes
**If Yes, complete the next two lines if this information is available**								
Diabetes duration (years)	2.00	2.50	0.00	2.00	2.00	0.00	2.00	0.00
Glucose-lowering therapy at time of death[c]	Yes: insulin	Yes: Humalog and Lantus	None at home reportedly; insulin IV drip upon admission	Yes: Lantus and Novolog	Yes: Lantus and Novolog	No	Yes: Humalog and Lantus	No
**RECOMMENDED INFORMATION**								
Donor cause of death								
Warm ischaemia time (h)								
Cold ischaemia time (h)								
Estimated purity (%)								
Estimated viability (%)								
Total culture time (h)[d]								
Glucose-stimulated insulin secretion or other functional measurement[e]								
Handpicked to purity? Please select yes/no from drop down list								

Additional notes								

**Islet preparation**	**105**	**106**	**107**	**108**	**109**	**110**		
**MANDATORY INFORMATION**								
Unique identifier	6405	6418	6435	6472	6473	6477		
Donor age (years)	29.1	24.86	24.75	10.25	13.21	19.87		
Donor sex (M/F)	Female	Male	Female	Female	Male	Female		
Donor BMI (kg/m^2)	42.5	26.4	26.9	16.6	17.9	25.3		

Donor HbA_{1c} or other measure of blood glucose control	7	7.5	11.6	9.7	12.8	10.9		
Origin/source of islets[b] Please see authors' note: N/A								
Islet isolation centre Please see authors' note: N/A								
Donor history of diabetes? Please select yes/no from drop down list	Yes	Yes	Yes	Yes	Yes	Yes		
**If Yes, complete the next two lines if this information is available**								
Diabetes duration (years)	0.60	11.00	14.75	4.00	6.00	8.00		
Glucose-lowering therapy at time of death[c]	Yes: insulin	Yes: insulin	Yes: Insulin and Tresiba	Yes: insulin	Yes: Lantus	Yes: Lantus, Humalog, Insulin aspart/ degludec		
**RECOMMENDED INFORMATION**								
Donor cause of death								
Warm ischaemia time (h)								
Cold ischaemia time (h)								
Estimated purity (%)								
Estimated viability (%)								
Total culture time (h)[d]								
Glucose-stimulated insulin secretion or other functional measurement[e]								
Handpicked to purity? Please select yes/no from drop down list								

Additional notes								
